# Executive functions predict verbal fluency scores in healthy participants

**DOI:** 10.1038/s41598-020-65525-9

**Published:** 2020-07-07

**Authors:** Julia Amunts, Julia A. Camilleri, Simon B. Eickhoff, Stefan Heim, Susanne Weis

**Affiliations:** 1 Institute of Neuroscience and Medicine (INM-7 Brain and Behaviour), Research Center Jülich, Jülich, Germany; 2 Institute of Systems Neuroscience, Heinrich-Heine University, Düsseldorf, Germany; 3 Institute of Neuroscience and Medicine (INM-1 Structural and functional organization of the brain), Research Center Jülich, Jülich, Germany; 4 Department of Psychiatry, Psychotherapy und Psychosomatics, Medical Faculty, RWTH Aachen University, Aachen, Germany

**Keywords:** Neuroscience, Psychology

## Abstract

While there is a clear link between impairments of executive functions (EFs), i.e. cognitive control mechanisms that facilitate goal-directed behavior, and speech problems, it is so far unclear exactly which of the complex subdomains of EFs most strongly contribute to speech performance, as measured by verbal fluency (VF) tasks. Furthermore, the impact of intra-individual variability is largely unknown. This study on healthy participants (n = 235) shows that the use of a relevance vector machine approach allows for the prediction of VF performance from EF scores. Based on a comprehensive set of EF scores, results identified cognitive flexibility and inhibition as well as processing speed as strongest predictors for VF performance, but also highlighted a modulatory influence of fluctuating hormone levels. These findings demonstrate that speech production performance is strongly linked to specific EF subdomains, but they also suggest that inter-individual differences should be taken into account.

## Introduction

Executive functions (EFs) refer to a set of cognitive processes that allow for goal-directed behavior through the regulation of various cognitive subprocesses. Since EFs permeate behavior, they also impact daily activities as well as social and personal development, including school or job success^1^. The importance and pervasiveness of EFs has led different fields of study to investigate these control mechanisms with the goal of differentiating the various subdomains of EFs. This, in turn, has resulted in a number of different conceptualizations based on different approaches, all attempting to subdivide EFs into different domains. While a consensus does not yet exist about how exactly to subdivide and name EFs, there is general agreement that there are three core EFs: (1) cognitive flexibility, (2) working memory and (3) inhibition^1^ (but see Karr *et al*.^2,3^). Higher-order EFs, such as reasoning, planning and problem solving, are then built on the basis of these subdomains.

The various sub-domains of EFs have been shown to be impaired in a number of neurological and psychiatric diseases, such as attention-deficit/hyperactivity disorder (ADHD)^4^, Parkinson’s disease^5^, depression^6^ and schizophrenia^7^. Different diseases present their own typical EF deficits and clinical diagnosis attempts to assess the specific patterns of the disease. For example, in the case of Parkinson’s disease, patients suffer from difficulties in dual-tasking which is reflected in the deficient combination of memorizing and manipulation of thoughts and tasks^8^ but also in impaired speech characterized by semantic paraphasias and reduced word fluency due to a lack of EFs^5,9^. To assess these symptoms different test batteries have been developed. These batteries, which include tests tapping into the different EF sub-domains, are used for neuropsychological assessment in both clinical settings and lab-based environments. Commonly used batteries are the Delis-Kaplan Executive Function System (D-KEFS) and the *Vienna Test System*, both of which offer a wide range of tests probing each of the EF sub-domains and have been independently validated^10–12^. Commonly used tasks tapping into the different sub-domains of EFs comprise the *Wisconsin Card Sorting test* (WCST), *Tower of London* (ToL) and *Trail*-*Making* test (TMT) to assess cognitive flexibility^5,13,14^, *n*-*back* tasks and the *Corsi block tapping* test to cover the sub-domain of working memory^15,16^ and the *Stop*-*signal* task or the *Stroop* test (color-word interference) to probe the sub-domain of inhibition. All of these are commonly used in the clinical^5,17^ as well as in the scientific context^14,18^. Importantly, due to the overlap of the different domains of EFs, these tests cannot be assumed to target one specific domain of EFs only.

Beside subdomain-specific EF tests, clinical and research test batteries also include a speech-based task, namely the verbal fluency (VF) task but the explicit involvement of different EF subdomains in the VF task is reported controversially especially when considering inter-individual differences^19^. The well-established and often-used VF test assesses the number of words generated in a given time (usually 60 seconds) and has been found to be a sensitive measurement for testing EFs in both non-clinical groups^19^ as well as in neurological patients^20^. VF tests mainly comprise two types of tasks: The phonemic/lexical VF, requiring the generation of as many words as possible beginning with a specific letter (e.g. C, F); and semantic VF, in which the examinee is asked to produce words that belong to a specific semantic category (e.g. fruits, animals). Additionally, most VF tests include a switching task in which words from two different categories are produced in an alternating order^21^.

The relationship between VF and the various subdomains of EFs has frequently been investigated in both healthy controls^19,22–24^ and patients^17,25^. Concerning the relationship between VF and working memory, some studies showed that better working memory performance leads to less perseveration errors^26^ and a higher total score of produced words in the VF task^22,27^. However, a clear link between working memory and VF performance has so far not been found^19,28^. Similarly, the relationship between VF and response inhibition is not clear yet. While some studies report lower scores in VF concomitating with a decline of inhibition performance^22^ other studies failed to find a link between VF and inhibition^20,29^. Finally, regarding the relationship between VF and cognitive flexibility, studies report a positive correlation of switching between categories in VF tasks and cognitive flexibility performance^21^. However, there are also findings which indicate that there is no relationship between EFs and VF performance^30^.

Altogether, results concerning the relationship of EFs and VF are ambiguous. This might, at least partly, be based on inter-individual variability of both EFs and VF. For example, a pronounced effect of age was identified by multiple studies showing a significant negative correlation between age and the different aspects of EFs as well as VF performance^31–34^. Furthermore, fluid intelligence has been found to be related to the performance in EFs tasks tapping into the subdomains of planning and reasoning and^35^. In contrast, inhibition was shown to be independent of intelligence in children with problems performing attention tests^36^.

The complex involvement of EFs in VF performance has also been shown to be modulated by the influence of inter-individual variability like dynamically varying hormonal levels. Especially sex hormones like estradiol and progesterone have been shown to influence performance in EFs tasks^37,38^. It was shown that cognitive performance varies during the different phases of the menstrual cycle with high progesterone and estradiol levels leading to faster reaction times and better accuracy^37,39^. Moreover, cortisol, which is mostly associated with stress, appears to impact EFs, but literature addressing this topic is ambiguous. On the one hand, studies found a positive relationship e.g. between cortisol level and working memory^40^ or cortisol level and performance in cognitive flexibility tasks^41^ in men. On the other hand an inverse relationship was found in cognitive flexibility tasks in women^41^ and in working memory performance^42^. Additionally to the influence on EFs varying hormonal levels could be also linked to VF performance^43^. To investigate the role of varying hormonal levels studies implement different procedures: While some inject specific hormones and assess the change of cognitive functions due to this injection^44,45^ other studies analyze intra-individual differences measuring the naturally varying hormonal level at different points of time^37,40^. Test data of EFs and VF are commonly analyzed with classical statistical methods. For example, correlation analyses have been previously used to investigate the relationship of VF and specific subdomains of EFs^46^. Other studies have investigated group differences between patients and healthy controls to e.g. examine sex differences in VF strategies^19^ or to explain the relationship of memory and VF in patients with Alzheimer’s disease^28^. Furthermore, factor analysis has also been applied to investigate common cognitive structures of VF performance^24,30^.

Considering previous literature investigating the relationship of EFs and VF in more detail it is obvious that each work contributes to a better understanding of this relationship but generalizing this knowledge is still difficult. Specifically, these limitations are e.g. due to the small subject size or reduced EF test batteries which does not represent overall EF performance. Generalizability is also restricted due to the applied methods. All the above-mentioned methods are applied to investigate within-sample effects to understand the theoretical hypothesis-driven neuropsychological relationship between VF performance and EFs. However, it is so far unclear to what extend VF task performance reflects the different subdomains of EFs. To address this question, more advanced statistical methods should lead to a more detailed insight into the complexity of VF performance. Machine learning models can be used to characterize complex behavior with the ultimate goal of identifying and predicting psychiatric diseases^47,48^. In contrast to classical statistical analyses, these prediction analyses use large sample sizes and a high number of variables as well as a cross-validation approach by training a model on part of the dataset and then validating it on unseen data. Applying machine learning methods on a wide variety of EF tests enables to capture the complex and non-linear relationship of EFs and VF performance.

To contribute to a deeper understanding of the so far inconclusive relationship between EFs and VF, the present study used a machine learning approach to investigate to what extent VF performance can be explained by subdomain-specific EF tests. We hypothesize that VF performance can be explained by a conglomeration of cognitive flexibility, working memory and inhibition test scores, which is further modulated by individual variations of fluctuating hormone levels.

## Methods

### Participants

The age of the 253 healthy participants was ranging from 20–55 years (mean age 35.3 ± 11.0, 99 males). Participants were monolingual German speakers and received different levels of education (finished middle school: 10, professional school/job training: 70, finished high school with a university-entrance diploma: 76, university degree: 97). Participants were recruited in North Rhine-Westphalia (Germany) via social networks and the Forschungszentrum Jülich mailing list. Testing sessions took place at the Forschungszentrum Jülich, with a duration of 150–180 minutes depending on the time needed for instructions and the speed with which the participants passed the tests. A remuneration fee of €50 was paid.

### Data collection

Data was collected by four different examiners, all of whom conducted several pilot testings and were instructed by the study leader to ensure a common standard. The examiner gave standardized instructions before starting each test and help was provided by the examiner whenever the participant had any questions regarding the instructions or tests. The testing session included 13 EF tests and 3 semantic VF tasks. The EF test battery consisted of computerized versions of neuropsychological tests covering domains of inhibition, working memory and cognitive flexibility. Ten of these tests were taken from the *Vienna Testsystem* test battery and three were designed with *PsyToolkit*^49^. In this study, we assessed commonly used EF tests like the *Stroop* and *TMT*. We used a broad selection of EF tests to cover all subdomains of EFs and to detect most influencing tests and their variables. A complete list of the tests is shown in Table 1.

**Table 1:** Overview of executive function test battery.

Measure	Description	Main variables
**Cognitive flexibility**/**Planning**		
Trail-Making test	The task consists of 2 parts. In part A, numbers from 1–25 are displayed on the screen in a haphazard fashion. The task consists of clicking on the numbers in sequential order as quickly as possible. In part B numbers from 1–13 and letters from A-L are presented on the screen. participnts must click on the numbers and letters alternately and in ascending order.	Errors in part A/B, difference part B-A, quotient B/A
Raven’s Standard Progressive Matrices	Eight items that form one pattern are shown to the participants. The task requires the participants to identify one missing item out of 6 choices to complete the pattern. The difficulty of recognizing each pattern increases during the course of this test.	Process time, correct items
Wisconsin Card Sorting test	Four stimulus cards illustrating different geometrical figures are presented. These cards differ in the number, color and form of the figures. The task is to match one additional card to one of the four cards using the correct rule (match for number, colour or form of figure) without knowing which rule is applied.Thus, participants are required to shift rules accordingly.	Number of perseveration/non-perseveration errors
Tower of London	Three rods are presented on the screen: The left rod holds three balls, the middle rod two balls and the right rod one ball. The participants are asked to move the balls from the starting state to ta target position using a minumum number of moves.	Planning ability, number of correct respones
Cued task switching	A coloured figure is presented on the screen. Participants are required to respond to either the color or figure task. Figure task: Particpants press matching button (left or right) depending on the type of the figure (triangle or rectangle); colour task: Particpants press matching button (left or right) depending on the colour of the figure (blue or yellow).	Number of incongruent/congruent errors
**Working memory**/**Attention**		
N-back non verbal	A sequence of 100 abstract successive figures are presented to the particpants. The task consists of indicating whether the current stimulus matches the figure shown two turns back (2-back paradigm).	Number of correct and false responses
Non-verbal learning test	Nonsensical, irregular, and geometric figures are presented on the screen. In the course of the test some figures are shown multiple times. For each figure the participants has to decide whether the current figure has already appeared or whether this figure is being shown for the first time.	Correct/false responses, sum of difference between correct minus false responses, process time
Corsi block tapping test	Nine irregularly arranged cubes are presented to the participants. A cursor touches a certain number of cubes in a specific order; The task is to repeat the given sequence correctly. The length of the sequence increases the more correct sequences the particpants complete.	Block span, correct/false items, error types (omission, sequence mistake)
WAF-G (divided attention)	The participants are required to focus on two geometric figures and one auditory stimulus. At a certain interval the stimuli change their intensitiy (figure gets lighter and/or auditory stimulus gets higher). The participants have to respond when two stimuli become lighter/higher twice in a row.	Mean reaction time, number of false alarm, missed items
WAF-R (spatial attention)	Four triangles are presented in four spatial positions (similar to Posner paradigm). The participants are required to react if a triangle changes intensity (gets darker). In the neglect test a interfering/matching visual cue is given but this cue do not always indicate the correct answer.	Mean reaction time, number of false alarm, missed items
**Inhibition**		
Stop-signal task	The test consists of two parts: 1) The participants are asked to respond to the direction of an arrow stimulus. 2) The participants have to repeat task as in previous step but should withhold their motoric response whenever they hear an auditory signal.	Stop-signal reaction time, stop-signal delay, number of different error types
Simon task	The participants are asked to press the right button if they read the word "right" and the left button if they read the word "left". The words are either presented on the right or left part of the screen. The reaction time of the participants is usually longer whenever the stimlus is incongruent to its position (e.g. the word "left" is on the right side of the screen).	Interference reaction time, incompatible/compatible errors
Stroop test	Names of colors (e.g., "blue", "green", or "red") are displayed on the screen in a color which is not denoted by the name (i.e., the word "blue" is printed in red). The test consists of two conditions: 1) Naming - participants are asked to respond to the colour of the words; 2) Reading - participants are asked to respond to the meaning of the word with naming. A baseline measure is taken at the start of the test to assess reading and color naming (color and word refer to the same concept).	Baseline time of naming and reading, reading interference, naming interference, errors

Results of the neuropsychological tests can be seen in Table 2.

**Table 2:** Neuropsychological data of participants.

	Variable	M ± SD	Min - Max
Age		35.33 ± 11.04	20–55
Education		4.05 ± 0.90	2–5
Cortisol		0.12 ± 0.08	0.001–0.42
Estradiol		3.61 ± 5.27	0.01–44.7
Progesterone		65.09 ± 93.62	6.25–940.97
Testosterone		79.96 ± 99.5	2.41–597.61
Trail Making Test	Difference part A-B [sec]	7.60 ± 6.25	−3.32–40.57
Raven’s Progressive Matrices	Correct items	27.86 ± 3.3	14–32
Wisconsin Card Sorting Test	Perseveration errors	7.91 ± 3.45	4–24
Tower of London	Planning ability	7.51 ± 2.20	1–12
Cued Task-Switching	Switch costs (reaction time switch tasks - reaction time in non-switch tasks)	0.05 ± 0.08	−0.15–0.39
N-back nonverbal	Correct items	8.40 ± 2.94	1–14
Non-verbal learning Test	Sum of difference between correct minus false	19.54 ± 7.78	−4–35
Corsi Block Tapping Test	Block span	5.68 ± 1.10	3–9
WAF-G (divided attention)	False alarm (crossmodal)	3.10 ± 4.86	0–34
WAF-R (spatial attention)	Errors	3.61 ± 3.38	0–18
Stop-Signal Task	Stop signal reaction time (mean reaction time - mean stop signal delay) [sec]	0.21 ± 0.07	0.03–0.50
Simon Task	Reaction time difference (reaction time incongruent - reaction time congruent items) [sec]	0.03 ± 0.04	−0.14–0.16
Stroop Test	Reading interference [sec]	0.14 ± 0.08	−0.04–0.50
Stroop Test	Naming interference [sec]	0.13 ± 0.08	−0.02–0.46
Semantic Verbal Fluency sum1		36.77 ± 8.30	19–57
Semantic Verbal Fluency sum2		26.08 ± 6.60	11–45
Semantic Verbal Fluency sum3		21.98 ± 4.34	8–34
Semantic Verbal Fluency sum all	sum1 + sum2 + sum3	84.83 ± 15.45	50–125

The semantic VF tasks were based on the *Regensburger Wortflüssigkeitstest*^50^. This test is a standardized neuropsychological assessment that has been thoroughly tested for reliability, validity and objectivity^50^. Due to language-specific differences in the frequency and usage of letters and categories^51^ this German version of VF task was used. Two of the tasks were simple semantic VF tasks in which the participant had to name animals (t_1_) and jobs (t_2_). The third semantic VF task was a switching task in which the participant switched between fruits and sports (t_3_) within the same task. Each of the three tasks was performed for 2 minutes. The VF tasks were presented with *Presentation* software (*Neurobehavioural Systems*) and the participant’s responses were recorded automatically. Following the testing session, the recorded speech was transcribed and words were coded manually as being either correct answers or errors. The number of correct words were counted for each task (t_1_, t_2_, t_3_) and the sum score of total number of correct words across all three VF tasks was used in all further analyses. To broadly represent VF performance, the sum of all VF tasks was selected to include different aspects of the task. This variety of VF performance is beneficial to build a machine learning model which is complex enough to reflect the complex patterns of VF performance.

In addition to the main test set of EFs and VF tasks, phenotypical data was collected through questionnaires to gather information regarding the physical and psychological well-being of the participants. These questionnaires included the Beck Depression Inventory (BDI-II) (Beck, Steer & Brown, 1996) which was used to collect information regarding depressive symptoms. Saliva samples were collected at the beginning and at the end of the test session. The two saliva samples of each subject were sent to an external lab which pooled both samples before carrying out analysis for cortisol, progesterone, estradiol and testosterone. Additionally, the testing session also comprised further speech tests (word-picture interference task, picture description, spontaneous speech), for which results will not be reported here, as they will be independently analyzed. This additional data will then be described in a subsequent paper. Moreover, we aim to publish a data paper which will describe all aspects of data collection, test selection and testing procedure in detail while also making this data publicly available.

Collection and analyses of the data presented here was approved by the ethics committee of the Heinrich-Heine University Düsseldorf. We confirm that all experiments were performed in accordance with relevant guidelines and regulations. Moreover, informed consent was obtained from all participants.

### Data analysis

The original dataset of 253 participants was reduced to 235 due to missing data of some participants (94 males; 101 participants were aged between 20–31, 70 between 32–43, 64 between 44–55). From all EF tests 72 variables (Supplement 1) were extracted based on the features provided by the *Vienna Testsystem* and *PsyToolkit*^49^. VF performance was represented by the sum score of correct words across all VF tasks.

Two independent analyses were computed. In a first analysis, Spearman correlations were computed to analyze the relationship of each EF variable and VF sum scores. Here, a reduction of the 70 EF variables was used. Specifically, EF variables were selected based on the EF test manuals provided by the *Vienna Testsystem* in 10/13 EF tests. In cases where multiple main variables were provided by the *Vienna Testsystem*, the main variable was selected based on previous literature investigating EF performance. In contrast to the *Vienna Testsystem*, tests run within *Psytoolkit* are not standardized and thus do not come with associated test manuals. Thus, the selection of main variables of tests designed with *Psytoolkit* (3/13) were selected based on previous literature.

Considering the influence of sex and age on the performance in EF and VF tasks^19,22,34^ data were adjusted for these variables by linear regression and analyses were computed with the residuals.

In a second analysis, the possibility of predicting VF from EF scores was investigated by applying supervised learning via a sparse (relevance vector machine; RVM) and non-sparse (partial least squares; PLS) model using 72 EF variables (Supplement 1). Generally speaking, sparse models aim to reveal a sparse structure and detect correlations among redundant features^52^. Specifically, RVM is based on the Support Vector Machine (SVM) but is a Bayesian sparse technique which allows for the prediction of a specific target value from a set of different features. In contrast, PLS is similar to principal components regression and is based on covariance. Results given in the main manuscript focus on the RVM approach, while results for the PLS analysis are given in the supplement. Sex and age were regressed out from VF score and from EF data in a cross-validation consistent way.

Before running the prediction analysis, data was transformed to z-scores. A 10-fold cross-validation was then performed for which the data set was randomly split into 10 sets, 9 of which were used for training while the 10^th^ set was held back and used to perform the prediction in previously unseen data. Ten replications of the 10-fold cross-validation were performed and thus 100 prediction models were computed. Prediction performance was assessed by computing the correlation between real and predicted values.

Beside testing statistical significance of prediction performance, we also examined which specific EF features significantly impact prediction performance. To determine which EF features (EF test variables) contribute most strongly to the prediction, we employed an approximate permutation test procedure, in which associations between features (total set of EF variables) and labels (VF sum score of each participant) were randomized. That is, the VF performance score was randomly permuted while the feature matrix was kept unchanged. The RVM analysis was repeated for each permutation and accuracies for 100 permutations were used to construct an empirical null distribution for each feature, which was used to compute the statistical significance of the contribution of each feature as the proportion of permutated labels achieving a better prediction than then original labels.

## Results

### Correlations between executive function scores and verbal fluency performance

**Figure 1:**
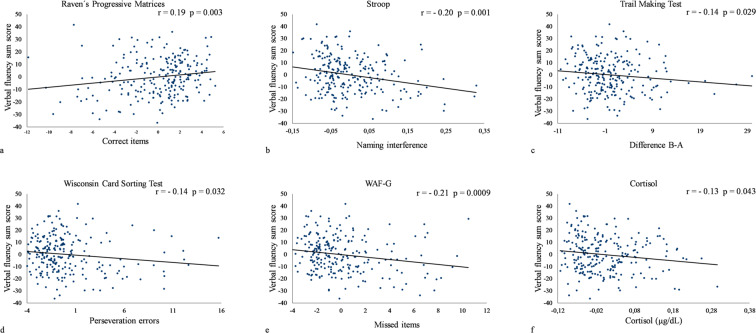
Plots of significant correlations of executive function tests and total verbal fluency sum score. The performance in verbal fluency task is represented by the total number of correct words produced across all three semantic VF tasks (t_1_ + t_2_ + t_3_).The negative correlation in plot b-f are due to the divergent direction of the scores since these variables describe different types of errors, reaction or process times (the higher the worse the performance) while the performance in the verbal fluency is represented by the total amount of correct items (the higher the better).

The correlation analyses identified multiple significant results which are shown in Fig. 1.

The highest negative correlation coefficient can be seen between the number of missed items in *WAF*-*G* and the VF performance (r = −0.21; *p* = 0.0009) indicating that a better performance in divided attention is associated with a higher VF score. Likewise, inhibition ability measured by the naming interference variable of the *Stroop* test (r = −0.20; *p* = 0.001) shows a negative correlation with the VF score. This result indicates that participants who successfully inhibited proponent behavior in the *Stroop* task perform better in the VF task. Additionally, abstract reasoning assessed with the *Raven’s Progressive Matrices* test (SPM) reveals a positive correlation (r = 0.19; *p* = 0.003) to VF performance indicating a demand of cognitive flexibility and planning while generating words from a specific category. Similar results were found for the *TMT* (r = −0.14; *p* = 0.029) and the number of perseveration errors in the *WCST* (r = −0.14; *p* = 0.032) which particularly reflect the involvement of cognitive flexibility and working memory in the VF task. Additional to the EF battery we also found a significant negative correlation of the VF tasks and the Cortisol level of the subjects (r = −0.13; *p* = 0.042).

### Prediction of verbal fluency performance from EF scores

**Figure 2:**
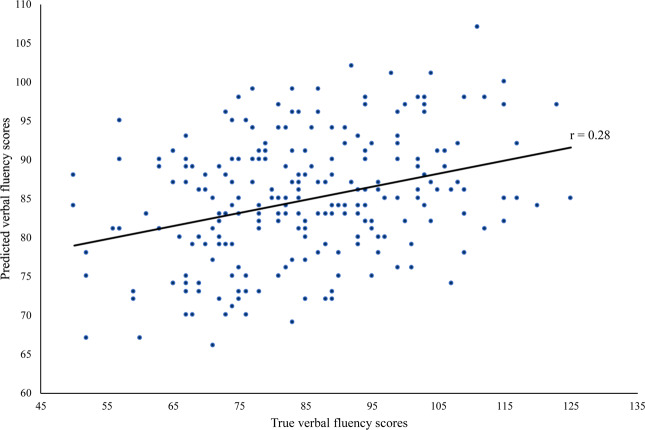
Correlation of true and predicted verbal fluency sum scores applying Relevance Vector Machine algorithm.

The correlation of true and predicted values was r = 0.28 (*p* < 0.0001) (Fig. 2).

**Figure 3:**
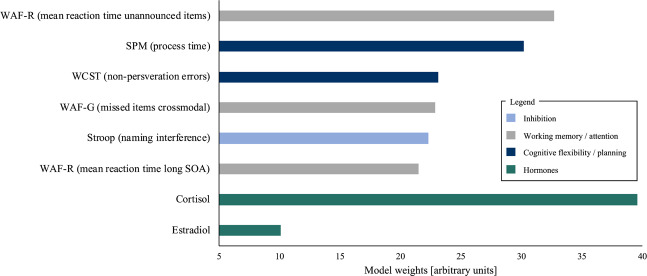
Features displaying strongest impact on prediction analysis.

In order to quantify the contribution of the different EFs variables to VF performance, features with significant model weights in the approximate permutation test were identified. As can be seen in Fig. 3, 8 features belonging to 4 different EF tests and 2 hormones were identified.

The EF feature with the highest impact on the prediction analysis is “mean reaction times” of unannounced items of the spatial attention test *WAF*-*R*. The RVM analysis also revealed another “reaction time” feature of *WAF*-*R* which represents “reaction time” in items with a long stimulus onset asynchrony. The influence of attention on VF performance was also shown in a feature of *WAF*-*G* assessing the number of missed items in a divided attention test. These results show that participants reacting faster in attention tests also perform better in the VF task, identifying overall reaction speed and correctness as a central component in VF performance. Since *WAF*-*R* is not only assessing attention but also includes inhibitory requirements, these results highlight the role of attention and inhibition during VF performance. The explicit role of inhibition can be also detected in the variable “naming interference” of the *Stroop* test indicating that inhibition is an essential component to successfully produce words within or between two different categories. The analysis also revealed the predictive meaningfulness of cognitive flexibility and planning, by showing that “non-perseveration errors” in *WCST* and “process time” in *SPM* contribute essentially to the prediction analysis. The *WAF*-*R* was the only test presenting more than one variable represented in the most predictive features, both of which contain reaction time information. With regards to non-EF features, the RVM analysis also identified stress hormone cortisol and sex hormone estradiol as highly predictive variables (Fig. 3).

Corresponding results of the PLS analysis revealed a correlation of true and predicted values of r = 0.35 (*p* < 0.0001). However, in contrast to the results of the RVM analysis, approximate permutation test did not reveal any significant *p*-values identifying specific EF features. Detailed results of the PLS analysis are given in the Supplementary Material (Supplement 2).

## Discussion

The aim of the study was to elucidate to what extent VF performance can be explained by different subdomains of EFs and which types of EF variables contribute most strongly to the prediction of VF performance. In a first step, we correlated the different EF scores with the number of correctly produced words across the three semantic VF tasks. This analysis revealed significant correlations between *SPM*, *Stroop*, *TMT*, *WCST*, *WAF*-*G*, *WAF*-*R* and the VF task performance. These EF tests tap into two EF domains, namely cognitive flexibility and inhibition. We further investigated the relationship of EF scores and VF by prediction analyses to gain insight into the contribution of the different EF test variables. We showed that EF data predict VF performance and that beside cognitive flexibility and inhibition, reaction time and attention play important roles in predicting VF performance. Additionally, hormonal influences were identified as meaningful parameters to predict VF performance, highlighting the influence of inter-individual differences in VF performance. We first discuss the results in the direct context of the different EF subdomains cognitive flexibility, inhibition and working memory. Secondly, the involvement of EFs in the VF task is discussed in a more general context addressing the role of attention as well as the meaningfulness of reaction times. Finally, the influence of varying hormonal levels illustrates the impact of inter-individual differences on VF performance.

Multiple tests within the domain of cognitive flexibility were shown to be related to VF performance. The highest correlation was found for the *SPM* test followed by *TMT* and *WCST*. While the correlation analysis revealed a relationship of these tests with VF performance, the prediction analysis confirms the importance of the features describing errors in *WCST*. Additionally, the prediction analysis highlights the component of processing speed during *SPM* which was not identified by correlation analysis. In congruence with these results, previous studies have linked VF with cognitive flexibility^21,53,54^. Paula *et al*.^21^ investigated this relationship in healthy adults, using simple and switching semantic VF tasks and three different EF tests, including the *TMT*. They found that this particular measure of cognitive flexibility correlated well with both simple and switching VF tasks. The influence of cognitive flexibility on VF was also examined in a study by Troyer *et al*.^54^ who discussed the importance of cognitive flexibility assuming that two different abilities are needed for VF: (1) verbal memory for the creation of clusters and production of words belonging to a specific subcategory; (2) strategic search and cognitive flexibility which enables shifting between clusters^54^.

It should be noted that the present results concerning the *SPM* might have to be treated with caution since this test also encompasses aspects of fluid intelligence^55–57^. Due to the relationship between EFs and fluid intelligence^58,59^, it may not surprise that fluid intelligence also impacts VF performance as has been shown in studies on schizophrenia^60^ and bipolar disorder patients^61^ as well as healthy controls^60^.

Altogether, considering that three out of five cognitive flexibility tests contribute to VF performance, the present results point to a crucial influence of cognitive flexibility on VF performance, especially to cluster words and switch between categories.

In addition to the domain of cognitive flexibility, inhibition tests were also identified to play a role in VF performance. Specifically, both the correlation analyses and prediction analysis revealed that the naming interference of the *Stroop* test is related to VF. Previous studies report ambiguous results when investigating the relationship between inhibition and speech production. For example, a positive correlation between inhibition, assessed with a stop-signal task, and the reaction time in picture naming^46^ was found but could not be validated in VF tasks^23^. Discussing these ambiguous results, the authors suggest that while stop-signal tasks measure the participant’s ability stopping a planned response (*response inhibition*), VF tasks tend to involve the ability of suppressing the activation of competitive target responses (*selective inhibition)*. In the present study selective inhibition was assessed by the *Stroop* test. In the naming subtask of the *Stroop* test the participant is asked to name the color in which the word is printed. Incongruent items, in which the color of the word does not match the written word evoke a longer reaction time, indicating that prepotent responses (i.e. the meaning of the written word) have to be suppressed. This is very similar to the kind of inhibitions that participants are challenged with in the VF task when needing to suppress words which have already been produced. In accordance with previous literature^24^, the present suggests that selective inhibition, specifically as reflected in the *naming interference* of the *Stroop* test, is a key parameter to drive VF performance. An alternative interpretation of the *naming interference* of the *Stroop* test and the total number of words produced in the VF task relates to the association between verbal processing speed and dominance of word reading. Individuals with a high verbal processing speed can be assumed to also show a stronger dominance of word reading then those with slower verbal processing. This stronger dominance of word reading, in turn, can be expected to go along with a stronger interference effect in the *STROOP* task, thus explaining the correlation between naming inference in the *STROOP* task and VF performance.

In addition to cognitive flexibility, working memory and inhibition we also investigated the role of attention in VF performance. While divided attention (*WAF*-*G*) was linked to VF performance in the correlation analysis, multiple variables of the spatial attention test (*WAF*-*R*) and divided attention test (*WAF*-*G*) contributed to VF performance in the prediction analysis.

Previous studies also described attention as a crucial cognitive function to perform VF^54^. In particular, it could be shown that divided attention particularly impacts the switching component of the VF tasks. At first glance, the influence of the spatial attention test on the speech task might be surprising since there are no spatial requirements in the VF task. Nevertheless, we assume that beside the component of attention *per se* the involvement of inhibition which is also part of this task might also have an effect on these results. With respect to the relationship of VF and divided attention, results are consistent with previous literature highlighting the influence of divided attention especially in the VF switching task^54^. To sum up, attention might be a crucial aspect for performing VF task. In particular, we hypothesize that attention is a fundamental and permanent cognitive requirement during updating the current status of already produced words as well as being efficient in producing words within or between two.

Surprisingly, the present study did not find a relationship between working memory and VF performance in both the correlation and prediction analysis. However, previous studies investigating the involvement of working memory in VF tasks also report ambiguous results: For example, one study assessing a digit-span and spatial-span test did not find a significant relationship between working memory and VF^28^. However, other studies have reported results that indicate updating of information and working memory performance have a high impact on VF scores^23,62,63^. Additionally, another study found the relationship between memory performance and VF to be specific to women^19^. The missing link between working memory and VF in the current study might be the type of variable which was selected to represent VF performance. Here, the sum of correctly produced words was used as the main measure of VF performance. While measuring VF performance, this variable does not contain information about the types of errors that occur during the VF task. Although word repetitions (*perseveration errors*) did not count as correct produced words this error type is not analyzed separately. However, perseveration errors are described as a sensitive indicator of working memory performance. Additionally, the relationship between working memory and VF performance measured with the total sum of words has so far been mainly investigated in patients or older participants^22,27^ but rarely in healthy controls. Thus, we assume that the number of correct produced words might be less meaningful to reflect working memory in healthy controls than error-specific parameters like perseveration errors.

In general, prediction results reveal specific EF tests and variables which are closely linked to VF performance. Besides differences in the strength of the relationship between certain EFs and VF, the EF test constructs themselves might also have partially influenced analysis. In particular, the reliability of some EF tests is discussed controversially^64,65^. Thus, some EF tests might not represent actual EF performance well and such a poor reliability of EF test might be reflected in the relationship of EF to VF as studied here.

Comparing correlation and prediction analyses, crucial differences in the results were observed. At first glance, the EF tests identified in the prediction analyses are similar to those of the correlation analyses but include additional variables. Specifically, the prediction analyses reveal a number of additional variables that measure how fast participants completed the tests and how many errors they made. While there is limited literature about the influence of processing speed in cognitive tasks on VF performance, some studies have addressed processing speed in general in the context of cognitive functions^66,67^. Another study found relationships between processing speed, working memory, inhibition and VF scores^24^. Additionally, poorer processing time has been associated with poorer cognitive performance in older adults^66^. The association between processing speed and EFs has been also observed in patients with depression^67^. In line with these findings, another study investigated the role of processing speed in schizophrenia patients and suggest that especially in working memory tasks assessing speed might be helpful to detect patterns of schizophrenia^68^. In respect to VF performance, processing speed was identified as being closely related to speech production^31^ and is reported as a predictor for VF in ageing^69^. Based on previous literature and our present findings, we assume that processing speed is a general aspect involved in both EFs tests and VF tasks. Particularly, it can be assumed that due to the time limit of 2 minutes in the VF tasks participants are zealous to name as many words as possible. This general behavior might also be relevant in EF tests. Thus, we suggest that processing speed and reaction times indicate that people acting fast in cognitive tasks also perform more successfully in VF tasks than participants thinking more in detail about their answer. Additionally, we assume that the complex influence of processing speed on VF performance might be beyond what can be described as a linear relationship. This might explain why the impact of speed is detectable in the prediction computation but was not found the correlation analysis.

In addition to the relationship between EFs and VF this study also assessed the influence of hormonal fluctuations to investigate the influence of inter-individual differences. The results showed that the stress hormone cortisol and the sex hormone estradiol have a high impact on VF performance. In line with other studies^44,45^ our analyses indicates that there is a negative correlation between cortisol level and the performance in cognitive functions. However, previous studies have also linked an increase of cortisol to better cognitive performance^40,41^. To our knowledge, rather little is known about the influence of estradiol on VF performance. However, studies investigating the influence of estradiol on EFs show that higher estradiol levels particularly leads to better performance in shifting and cognitive flexibility tasks^37,39^. Moreover, a link between hormonal contraceptives and VF performance^43^ has been shown. These results demonstrate that women taking hormonal contraception and consequently having significantly lower estradiol and progesterone levels, perform worse in the VF task than the control group^43^. The high impact of cortisol and estradiol in the prediction analysis suggest that fluctuating hormones are essential parameters for predicting VF performance and that intra-individual differences in hormone levels need to be considered when examining the relationship of cognitive functions and speech production tasks. Thus, it shows that although EF test variables are closely related to VF performance VF is a complex construct which is also driven by hormones and attention.

Our prediction analyses yielded important insights into the relationships between EFs, VF and inter-individual differences. However, some open questions remain concerning both inter-individuality and speech related topics. Firstly, due to the fact that inter-individuality influences both EFs and VF performance, further studies would benefit from gathering additional inter-individual parameters. For example, a test for assessing intelligence might be useful to control for the influence of intelligence on each test, especially on the *SPM*, making it possible to better differentiate the impact of cognitive flexibility on VF performance. Secondly, intra-individual differences could be further investigated by gathering saliva samples at two different time points. In this study saliva samples of each participant were pooled. A comparison of the hormones level before and after testing might help to provide insights into individual strategies dealing with stress. Beside inter-individual influences of hormonal levels on EFs^41^, studies also report intra-individual variety e.g. due to different phases of the menstrual cycle^38^. Therefore, an analysis of hormonal levels within each participant taken at different time points could reveal an additional dimension representing intra-individual differences.

Considering speech-specific issues, a vocabulary test could contribute to better understand inter-individual differences. Previous studies showed that the vocabulary size has a positive impact on VF performance^54,70^. Moreover, additional parameters reflecting VF performance could help to gain deeper insights of searching strategies during VF tasks. In particular, semantic analyses provide details of clustering and switching^24^ and could indicate the participant’s strategies which could then be linked to EF performance.

A more general consideration is related to the predictive methods as used in this study. An independent data set assessing the same variables that were used in the study does not yet exist. Thus, it was not possible to validate our results in a totally independent dataset. Instead, we applied 10-fold cross-validation by repeatedly training the model on parts of the data while keeping a subset out as a validation sample. However, we are aware of the need to validate our results in an independent dataset to better generalize our results and suggest a replication of this study on an independent sample which could prevent study-specific biases. However, due to the broad and specific collection of the EF test battery finding a similar data set could be difficult. An additional independent dataset with similar EF tests could be used to test split-half reliability investigating the construct of EF tests. Due to the high number of participants which is needed to apply machine learning methods it was not possible to split our data in two groups and running the prediction analysis on the split data. The ambiguous results of the RVM and PLS analysis also need to be considered. While both approaches revealed a significant correlation between true and predicted values, the PLS approach did not identify any significant features (Supplement 2). This might be due to the fact that PLS is a non-sparse machine learning method, which will include all features in the prediction model. In contrast, it is the nature of sparse models like RVM to build the prediction model based on most relevant features only.

Due to the high number of participants and the large battery of EF tests this study provides a detailed view on the involvement of EFs in VF tasks and examines the influence of fluctuating hormones. It investigated to what extent EF tests can represent semantic VF performance and shows that cognitive flexibility and inhibition are the main domains involved in performance on the VF task. Additionally, attention seems to be a central component of the VF task. The most striking observation to emerge from the data analysis was the new and more detailed view of the EF tests and variables that are best at predicting VF performance. While correlation analyses provided first insights into the relationship of EFs and VF, the prediction analyses revealed the importance of speed parameters. In particular, our results suggest that beside the influence of specific EFs, more general components such as attention and speed are crucial aspects of successful VF performance. These results also highlight the advantage of the prediction analysis since it revealed concrete variables of EF tests which also represents cognitive abilities not directly linked to specific EF subdomains or representing standard variables.

A better understanding of the cognitive demands that are required for the successful performance of VF tasks can potentially lead to a more wide-spread use of VF tests in the clinical context, thus EF tests that tend to be time-consuming and inaccurate. Additionally, VF tests tend to better reflect real-life conditions than lab-based EF batteries. A detailed knowledge of meaningful test variables could later on lead to insights into which subdomains of EFs could be replaced by VF tasks and which subdomains of EFs still have to be assessed by additional EF tests. This link between EF and VF represents a first step towards a speech-based EF-test. Furthermore, it indicates that in investigating the relationship of EF and VF the complex construct of VF performance should be considered in research and clinical context.

Furthermore, taking the influence of varying hormonal levels into account our study suggests that beside inter-individual differences intra-individual fluctuations could play an important role in evaluating VF performance in clinical context.

## Supplementary information

Supplementary Information.
