# Supplementary material for: Executive functions predict verbal fluency scores in healthy participants
Source: Sci Rep. 2020 Jul 7;10:11141. doi: 10.1038/s41598-020-65525-9 (PMC7341845; doi:10.1038/s41598-020-65525-9)
Supplement: Supplementary file 1 — Supplementary Information. [file 41598_2020_65525_MOESM1_ESM.docx]

**Executive functions predict verbal fluency scores in healthy participants**

Julia Amunts^1,2^, Julia A. Camilleri^1,2^, Simon B. Eickhoff^1,2^, Stefan Heim^3,4^ & Susanne Weis^1,2^

^1^ Institute of Neuroscience and Medicine (INM-7 Brain and Behaviour), Research Center Jülich, Jülich, Germany

^2^ Institute of Systems Neuroscience, Heinrich-Heine University, Düsseldorf, Germany

^3^ Institute of Neuroscience and Medicine (INM-1 Structural and functional organization of the brain), Research Center Jülich, Jülich, Germany

^4^ Department of Psychiatry, Psychotherapy und Psychosomatics, Medical Faculty, RWTH Aachen University, Aachen, Germany

Corresponding author:

Julia Amunts

Institute of Neuroscience and Medicine (INM-7 Brain and Behaviour), Research Center Jülich, Jülich, Germany

Wilhelm-Johnen-Str.

52428 Jülich

GERMANY

Correspondence to [j.amunts@fz-juelich.de](mailto:j.amunts@fz-juelich.de)

**Supplementary material**

**Supplementary Table S1: Overview of executive function test variables**

**Description of Partial Least Squares computations**

Partial least squares regression (PLS) is an extension of the multiple linear regression model and is closely related to principal components regression ^73^. The aim of this method is to find a linear regression model which describes the relationship between dependent and a set of predictive variables. Additionally, PLS creates weight relations that capture most information of the predictive variables. In particular, the dimensionality of the regression problem gets reduced while using fewer components than the original number of variables ^74^. Applying PLS in this study, EF scores were used as a set of features to predict the VF sum score label. Similar to the RVM computations both the correlation of true and predicted values and the influence of the most predictive variables were analyzed.

**Results of Partial Least Squares computations**

The correlation of true and predicted values was r = 0.35 (*p* < 0.0001) (Figure S2).

**Supplementary Figure S2: Correlation of true and predicted verbal fluency sum scores applying Partial Least Squares algorithm**

In order to quantify the contribution of the different EFs variables to VF performance, features with significant model weights should get identified. Applying approximate permutation test none of the features revealed a significant *p* value (Table S3).

**Supplementary Table S3: Overview of permutation test results after applying Partial Least Squares regression**
